# Mechanistic Target of Rapamycin (mTOR) in the Cancer Setting

**DOI:** 10.3390/cancers10060168

**Published:** 2018-05-30

**Authors:** James T. Murray, Andrew R. Tee

**Affiliations:** 1 School of Biochemistry & Immunology, Trinity Biomedical Sciences Institute, Trinity College Dublin, Dublin 2, Ireland; james.murray@tcd.ie; 2 Division of Cancer and Genetics, Cardiff University, Heath Park, Cardiff CF14 4XN, UK

**Keywords:** mTOR, cancer

## Abstract

This special issue on mammalian target of rapamycin (mTOR) explores the importance of mTOR in cell growth control and cancer. Cancer cells often exploit mTOR as a mechanism to enhance their capacity to grow. While protein synthesis is by far the best-characterized mTOR-driven process, this special issue also describes a wider array of mTOR-driven biological processes that cancer cells benefit from, including autophagy, cell cycle control, metabolic transformation, angiogenic signaling, and anabolic processes such as nucleotide biosynthesis and ribosomal biogenesis. Other areas of mTOR signaling covered in these reviews delve into cell migration, inflammation, and regulation of transcription factors linked to cancer progression.

## 1. Introduction

The six reviews in this special issue all describe the classical mTOR complex 1 (mTORC1) signaling pathway in cancer, involving nutrients, energy, and growth signaling inputs from mitogens and hormones. This review series also touches on mTOR complex 2 (mTORC2), which is beginning to emerge as being a prominent contributor to cancer progression. The mTORC2 complex also regulates a diverse range of signaling implicated in cancer progression, including the AKT serine/threonine kinase-related kinase and serum/glucocorticoid-regulated kinase 1 that is associated with carcinogenesis and chemoresistance mechanisms.

The review by Conciatori et al. focuses on the limited clinical success of using rapalogues (the drug inhibitor of mTORC1) as single agents to treat cancer, while combinatory drug treatments hold much more promise [1]. Combinatory drug targets and pathways are discussed, which include the angiogenic pathway, involving the vascular endothelial growth factor receptor and hypoxia signaling that drives metabolic transformation, survival, and metastasis. Also discussed is the combinatory therapy with mTOR inhibitors and bortezomib, where bortezomib is an indirect strategy to inhibit nuclear factor kappa B via blocking the proteasome. Also highlighted as a possible drug target is signal transducer and activator of transcription 3 (STAT3), involving inflammation and immunity and where signaling cross talk between mTOR and STAT3 is evident.

The review by Paquette et al. focuses on mTOR and autophagy, where there is growing evidence of its importance in cancer [2]. This is a detailed review describing lysosomal localized signaling mechanisms that sense converging signal inputs from nutrients, energy stress, and growth factors. There is controversy regarding the role of autophagy in cancer, described in this review as a ‘double-edged function of autophagy in tumor suppression and promotion’. Dysfunction of autophagy can promote cancer onset through cellular stress, while at later stages of cancer development the dependency of autophagy for the survival of cancer cells is less clear and greatly varies depending on the cancer.

Rad et al. define the cancer-promoting qualities of mTOR and delineate both mTORC1 and mTORC2 signaling in the cancer setting [3]. This review explores how mTORC1 orchestrates cell growth control and briefly considers protein translation with emphasis on protein synthesis of mTORC1-sensitive mRNA transcript as well as cell cycle control and metabolic transformation. This review also describes the current limitations of mTOR inhibitors in the clinic due to their ‘cytostatic’ nature, and the consequences of inhibiting mTORC2 complexes. However, with the emergence of second- and third-generation mTOR inhibitors and the feasibility of combinatory therapies that can further exploit tumor vulnerabilities, this review highlights future benefits to cancer treatments.

Wang et al. describe the central involvement of eukaryotic elongation factor 2 kinase (eEF2K) in cancer [4]. This is a detailed review on eEF2K, which is a negative regulator of protein synthesis and cell growth and is mTORC1-regulated. Paradoxically, eEF2K promotes cancer cell survival during conditions of nutrient deprivation as it protects cancer cells from endoplasmic reticulum stress. This review implicates eEF2K as a promising drug target to treat some cancers, such as breast and intestinal cancer. Other less characterized aspects of eEF2K biology are discussed, including its emerging role in migration and invasion.

The review by Faes et al. focuses on the anti-angiogenic properties of mTOR inhibitors and their clinical application to treat cancer [5]. The multifaceted role that mTORC1 has in the regulation of angiogenic signaling through hypoxia inducible factor is highly topical and is discussed in detail. Of importance, this review covers the current clinical challenge of drug resistance when using anti-angiogenic drugs. Highlighting a possible way forward, the authors explore combinatory radiotherapy with mTOR inhibitors as one possible strategy, where using mTOR inhibitors results in radio-sensitization and reduction in tumor blood vessels.

Finally, Viel et al. explore the role that mTOR has in natural killer (NK) cells, where they utilize mTOR to enhance their cellular metabolism and immune surveillance [6]. The central topic of this review is how the metabolic activity of NK cells could be enhanced clinically to improve their antitumor effector function(s). Cytokine signaling in NK cells that are influenced by mTOR include interleukin-15 and transforming growth factor-β and are explored in detail.

## 2. Conclusions

In conclusion, this review series focuses nicely on the most topical and relevant aspects of mTOR cancer biology, highlighting the significant advances made in both the basic mechanisms of mTOR signaling and its fundamental roles in governing the physiology of cell growth. These reviews also underscore the significant lack of knowledge in areas of mTOR biology that will be the focus of future research. In addition, these reviews cover the emerging themes of mTOR signaling in immune cells that are important for cancer immunosurveillence mechanisms and the clinical advances using next-generation mTOR inhibitors. Targeting mTOR and exploiting cancer vulnerabilities in drug combinations hold true promise for the treatment of a diverse array of cancers.

## Abbreviations

The following abbreviations are used in this manuscript:

eEF2K	eukaryotic elongation factor 2 kinase
mTOR	mechanistic target of rapamycin
mTORC1	mTOR complex 1
mTORC2	mTOR complex 2
NK	natural killer
STAT3	signal transducer and activator of transcription 3
